# Supplementary material for: Robust and Functional Immune Memory Up to 9 Months After SARS-CoV-2 Infection: A Southeast Asian Longitudinal Cohort
Source: Front Immunol. 2022 Feb 3;13:817905. doi: 10.3389/fimmu.2022.817905 (PMC8853741; doi:10.3389/fimmu.2022.817905)
Supplement: Supplementary file 1 [file DataSheet_1.pdf]

Supporting information

S1 Table. Cohort Description

	SARS-CoV-2-infected patients (n=64)
Age (y), median (min-max, IQR)	36 (12-75, 18.5)
Sex (%)	
Male	55 (86)
Female	9 (14)
Smoker (%)	8 (13)
Comorbidities (%)	
Diabetes	2 (3)
High blood pressure	7 (11)
Clinical spectrum (%)	
Asymptomatic	19 (30)
Symptomatic	45 (70)
Dysosmia and dysgeusia	15 (23)
Dyspnea	16 (25)
Fever	23 (36)
Cough or sore throat or running nose	35 (55)
Days from SARS-CoV-2 positive RT-PCR to sample collection, median (min-max)	
Acute	5 (2-9)
6-9 months	203 (166-258)
Time of SARS-CoV-2 RNA shedding (%)	
< 10 days positive	30 (47)
≥ 10 days positive	34 (53)

S2 Table. Monoclonal antibody list

Marker	Fluorochrome	Supplier	Catalog N.
C3/C3b/iC3b	APC	Cedarlane	CL7503APC
CCR6	BB515	BD Biosciences	564479
CCR7	APC	Biolegend	353214
CD107a	APC	BD Biosciences	560664
CD134	BB700	BD Biosciences	566559
CD137	PE-Cy7	Biolegend	309818
CD138	BV785	Biolegend	356538
CD19	APC/Cy7	Biolegend	302218
CD27	PerCP/Cy5.5	Biolegend	356408
CD3	BUV395	BD Biosciences	564001
CD3	Per-Cp5-5	Biolegend	317336
CD38	BV 605	Biolegend	303532
CD4	BUV496	BD Biosciences	612936
CD45RA	BV421	Biolegend	304130
CD56	BV421	Biolegend	362552
CD69	BV786	Biolegend	310932
CD69	PE-Cy7	Biolegend	310912
CD8	AF700	Biolegend	344724
CD8	APC-H7	BD Biosciences	560179
CXCR3	BV711	Biolegend	353732
IFN-γ	BV605	Biolegend	502536
IFN-γ	PE	Biolegend	506507
IgA	APC	Miltenyi Biotec	130-113-472
IgA	AF 647	Jackson ImmunoResearch	109-605-011
IgD	BV711	BD Biosciences	740794
IgG	APC	Life technology	A21445
IgG	FITC	Biolegend	410720
IgM	PE	Biolegend	314508
IgM	BV421	Biolegend	314515
IL-17	BV711	Biolegend	512328
IL-2	PerCP/Cy.5	Biolegend	500322
IL-4	BUV737	BD Biosciences	612835
IL-6	PE	Biolegend	501107
TNF-α	APC-Cy7	Biolegend	502944

A

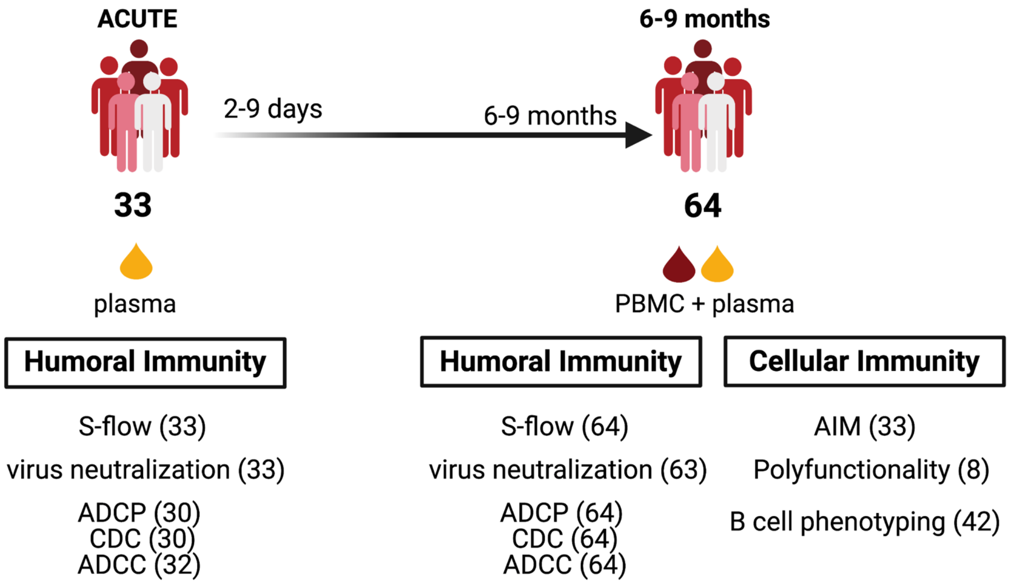

B

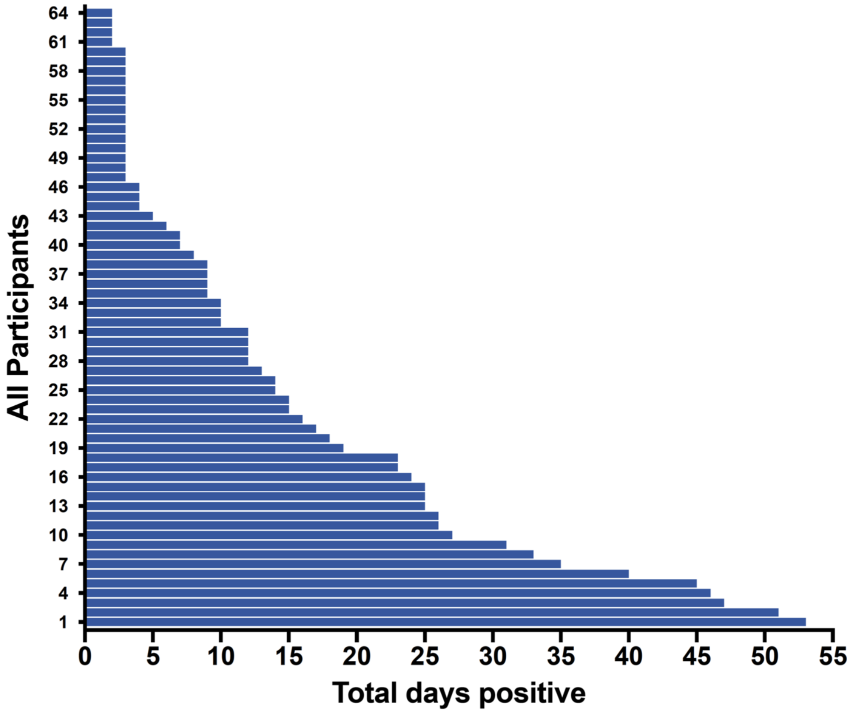

S1 Figure. Characterization of the cohort

(**A**) Graphical summary of the assays performed on samples of the different time points. (**B**) Length of RNA shedding in the patients.

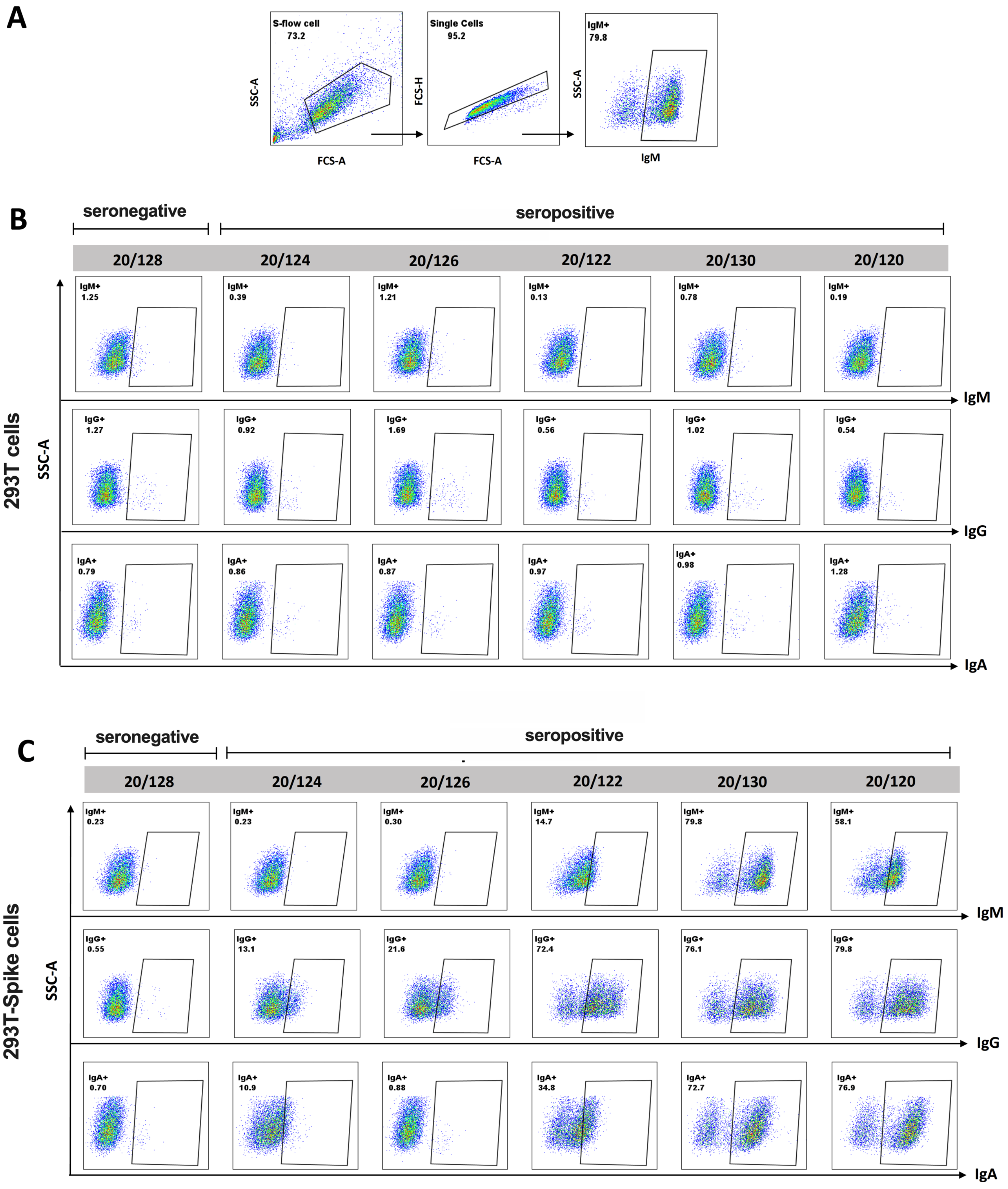
A
S-flow cell
73.2
SSC-A
FCS-A
Single Cells
95.2
FCS-H
FCS-A
IgM+
79.8
SSC-A
IgM
B
seronegative
seropositive
20/128
20/124
20/126
20/122
20/130
20/120
293T cells
SSC-A
IgM+ 1.25
IgM+ 0.39
IgM+ 1.21
IgM+ 0.13
IgM+ 0.78
IgM+ 0.19
IgM
IgG+ 1.27
IgG+ 0.92
IgG+ 1.69
IgG+ 0.56
IgG+ 1.02
IgG+ 0.54
IgG
IgA+ 0.79
IgA+ 0.86
IgA+ 0.87
IgA+ 0.97
IgA+ 0.98
IgA+ 1.28
IgA
C
seronegative
seropositive
20/128
20/124
20/126
20/122
20/130
20/120
293T-Spike cells
SSC-A
IgM+ 0.23
IgM+ 0.23
IgM+ 0.30
IgM+ 14.7
IgM+ 79.8
IgM+ 58.1
IgM
IgG+ 0.55
IgG+ 13.1
IgG+ 21.6
IgG+ 72.4
IgG+ 76.1
IgG+ 79.8
IgG
IgA+ 0.70
IgA+ 10.9
IgA+ 0.88
IgA+ 34.8
IgA+ 72.7
IgA+ 76.9
IgA

S2 Figure. Measurement of anti-S-binding antibodies using S-Flow

(**A**) Representative gating strategy (**B-C**) Percentages of spike-binding Ig were measured in plasma as percentage of cells, which are positive for anti-IgM, anti-IgG or anti-IgA. Specific binding was calculated as 100 x (% binding on S-expressing 293T cells - % binding on 293T control cells)/(100-% of binding to 293T control cells). Graph shows data from triplicate testing of the COVID-19 reference plasma panel (NIBSC 20/120, 20/124, 20/124, 20/126, 20/128, 20/130) obtained from the NIBSC.

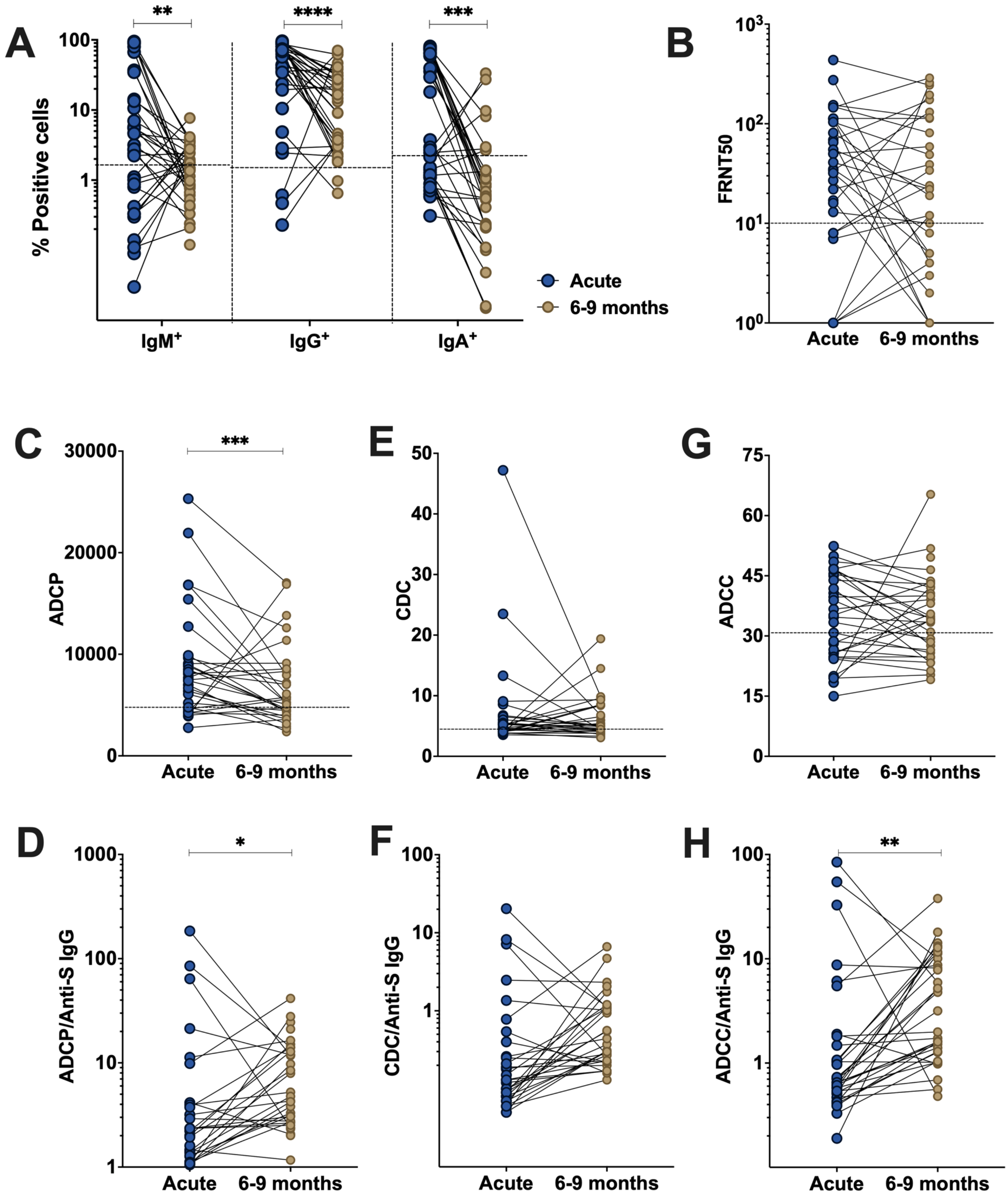
A
B
C
D
E
F
G
H
% Positive cells
IgM+
IgG+
IgA+
**
****
***
Acute
6-9 months
FRNT50
ADCP
CDC
ADCC
ADCP/Anti-S IgG
CDC/Anti-S IgG
ADCC/Anti-S IgG
*

S3 Figure. Analysis of antibody features in paired patient samples

Individuals were sampled 2-9 days post laboratory confirmation and 6-9 months later. (**A**) Amount of antibodies against spike protein were reported as percentage of spike-expressing 293T cells bound by IgM, IgG, IgA in the S-Flow assay. (**B**) SARS-CoV-2 neutralizing activity was calculated as FRNT50 titer in foci reduction neutralization test. (**C**) Comparison of ADCP activity in SARS-CoV-2-infected individuals in the acute phase of infection and 6-9 months post-infection. (**D**) Ratio of ADCP to anti-spike IgG measured by S-Flow. (**E**) Comparison of CDC activity in SARS-CoV-2-infected individuals in the acute phase of infection and 6-9 months post infection. (**F**) Ratio of CDC to anti-spike IgG measured by S-Flow. (**G**) Comparison of ADCC activity in SARS-CoV-2-infected individuals in the acute phase of infection and 6-9 months post infection. (**H**) Ratio of ADCC to anti-spike IgG measured by S-Flow. Statistical comparisons were performed by Wilcoxon test. The dashed line indicates the cutoff for positivity based on values calculated following formula: cut-off = % mean positive cells from 19 pre-pandemic samples + 3x standard deviation. Each dot represents result from a single individual. Lines represent median and IQR. Each dot represents one individual. Lines represent median and IQR. *$p < 0.05$, **$p < 0.01$, ***$p < 0.001$, and ****$p < 0.0001$. IgM/IgA/IgG antibody: n=33, Neutralization: n= 33, ADCP: n=30, CDC: n=30, ADCC n=32.

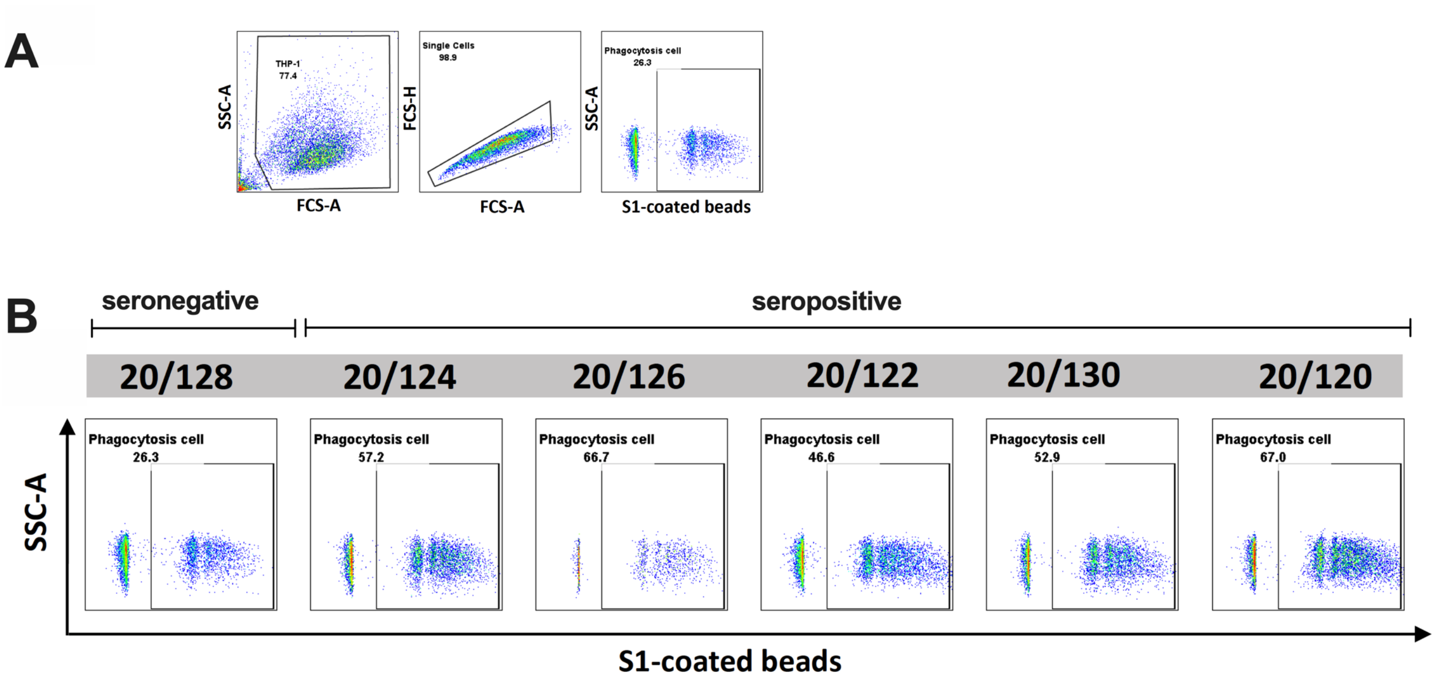

S4 Figure. Antibody-dependent cellular phagocytosis (ADCP) assay

Quantification of the S1-coated beads engulfed by THP-1 cells. (**A**) Representative gating strategy. (**B**) ADCP was defined by the percentages of THP-1 cells which are positive for FITC-neutravidin beads coated with biotinylated S1 protein. Representative ADCP assay using the COVID-19 reference plasma panel (NIBSC 20/120, 20/124, 20/124, 20/126, 20/128, 20/130) obtained from the NIBSC. The ADCP activity represents the integrate mean fluorescence intensity (iMFI) value (% positive fluorescence THP-1 cells x MFI of the positive fluorescence THP-1 cells). The experiment was performed in duplicate.

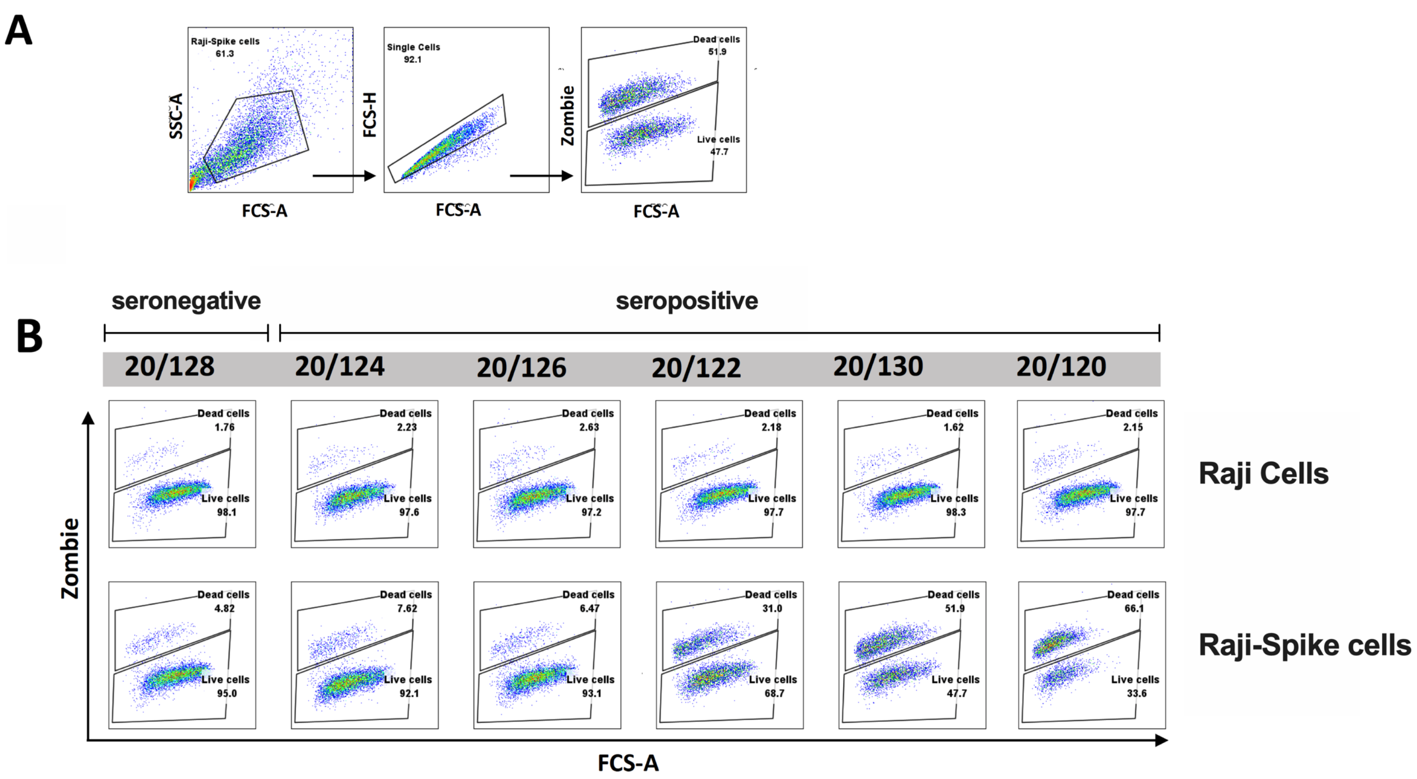

S5 Figure. Complement-dependent cytotoxicity (CDC) assay

SARS-CoV-2 plasma induces C3 deposition and cell death in spike-expressing Raji cells. (**A**) Representative gating strategy. (**B**) CDC assay was performed by using pike-expressing Raji cells as target cells, serum (pooled from 3 healthy donors) as complement source and heat-inactivated plasma from SARS-CoV-2 patients or from reference panel as antibody source. CDC activity was measured as the percentage of $C3^{+}Zombie^{+}$ pike-expressing Raji cells after incubation with plasma and complement. Representative CDC assay using the COVID-19 reference plasma panel (NIBSC 20/120, 20/124, 20/124, 20/126, 20/128, 20/130) obtained from the NIBSC. Complement-induced cell death was calculated as percentage of $C3^{+}$ dead cells of total cells using spike-expressing Raji cells as target cell. The experiment was performed in duplicate.

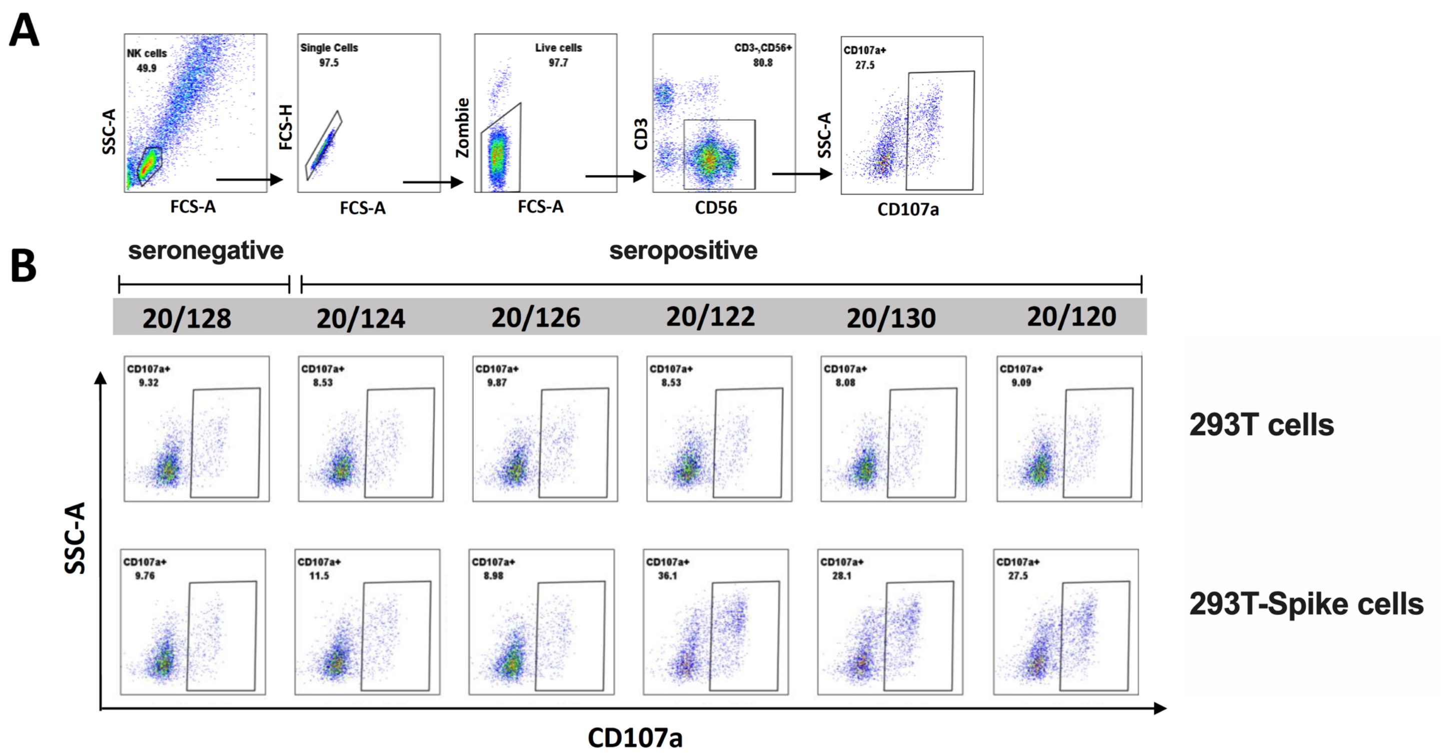

S6 Figure. Antibody-dependent cellular cytotoxicity (ADCC) assay

(**A**). Representative gating strategy. NK cells were identified by lymphocyte morphology, singlet, live cell and CD3^{-}CD56^{+}. The ADCC activity was defined based on NK cell degranulation (CD107^{+}). (**B-C**) ADCC activity of NK cells induced by incubation with COVID-19 reference plasma panel (NIBSC 20/120, 20/124, 20/124, 20/126, 20/128, 20/130) obtained from the NIBSC in the presence of 293T-spike cells or 293T control cells. The experiment was performed in duplicate.

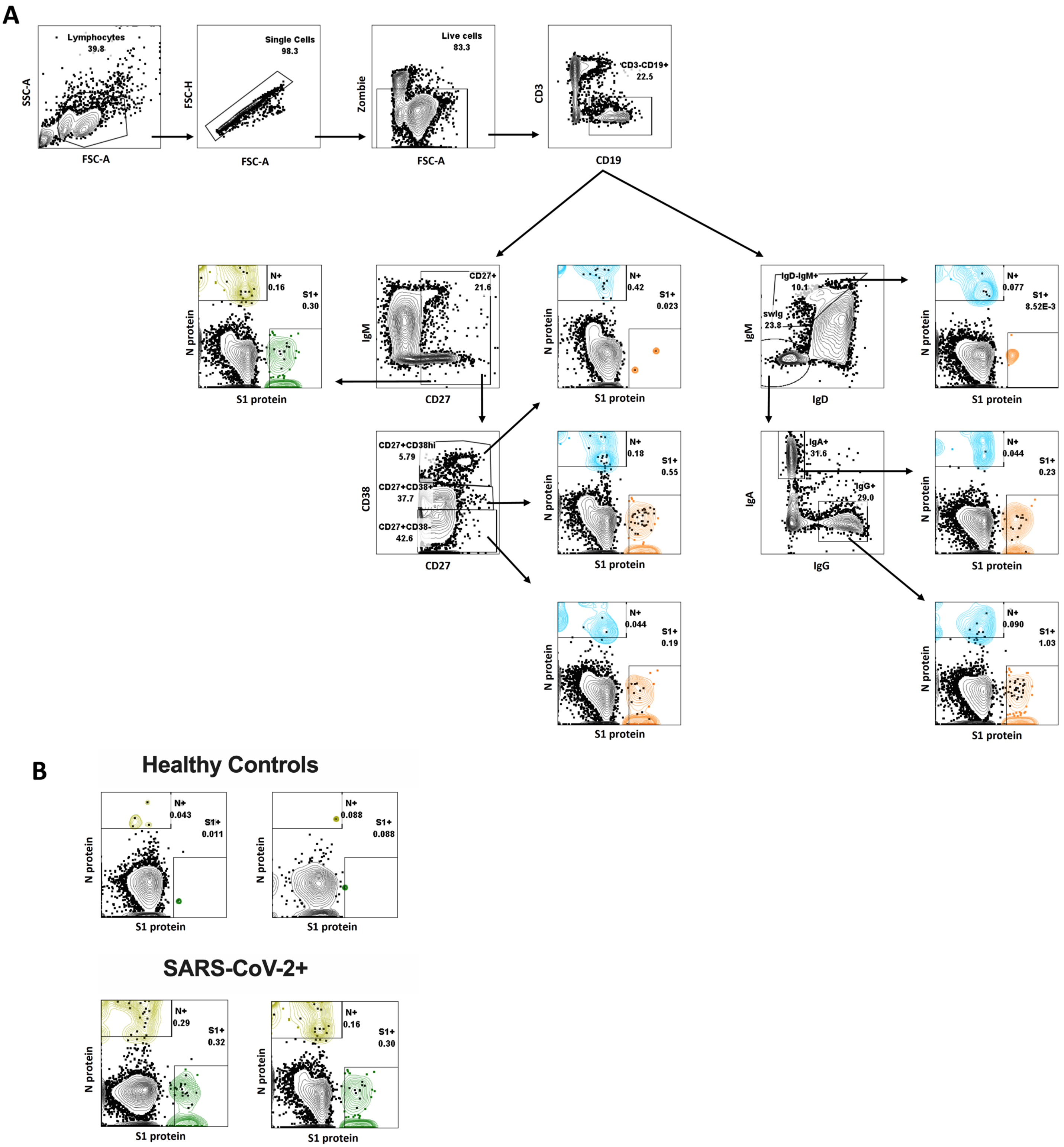
A
Lymphocytes
39.8
SSC-A
FSC-A
Single Cells
98.3
FSC-H
FSC-A
Live cells
83.3
Zombie
FSC-A
CD3-CD19+
22.5
CD3
CD19
N+
0.16
S1+
0.30
N protein
S1 protein
CD27+
21.6
IgM
CD27
N+
0.42
S1+
0.023
N protein
S1 protein
IgD-IgM+
10.1
swIg
23.8
IgM
IgD
N+
0.077
S1+
8.52E-3
N protein
S1 protein
CD27+CD38hi
5.79
CD27+CD38+
37.7
CD27+CD38-
42.6
CD38
CD27
N+
0.18
S1+
0.55
N protein
S1 protein
IgA+
31.6
IgG+
29.0
IgA
IgG
N+
0.044
S1+
0.23
N protein
S1 protein
N+
0.044
S1+
0.19
N protein
S1 protein
N+
0.090
S1+
1.03
N protein
S1 protein
B
Healthy Controls
N+
0.043
S1+
0.011
N protein
S1 protein
N+
0.088
S1+
0.088
N protein
S1 protein
SARS-CoV-2+
N+
0.29
S1+
0.32
N protein
S1 protein
N+
0.16
S1+
0.30
N protein
S1 protein

S7 Figure. Representative gating strategy used to define antigen-specific B cells

(**A**) B cells were defined from the gates of morphology of lymphocytes, singlets, viability and $CD3^{-}CD19^{+}$. Total B cells ($CD19^{+}$) were further gated for B cell subsets including resting memory B cells ($CD27^{+}CD38^{-}$), activated memory B cells ($CD27^{+}CD38^{+}$) and plasma blasts ($CD27^{+}CD38^{hi}$). S1-specific B cells and N-specific B cells were determined. Total B cells ($CD19^{+}$) were further separated into non-class-switched mature cells ($IgD^{-}IgM^{+}$) and class-switched $IgM^{-}IgD^{-}IgA^{+}$ and $IgM^{-}IgD^{-}IgG^{+}$ cells. The S1-specific B cells and N-specific B cells were determined. (**B**) Representative image comparing S1- and N-specific B cells in 2 different healthy controls and 2 different SARS-CoV-2-infected patients 6-9 months post infection.

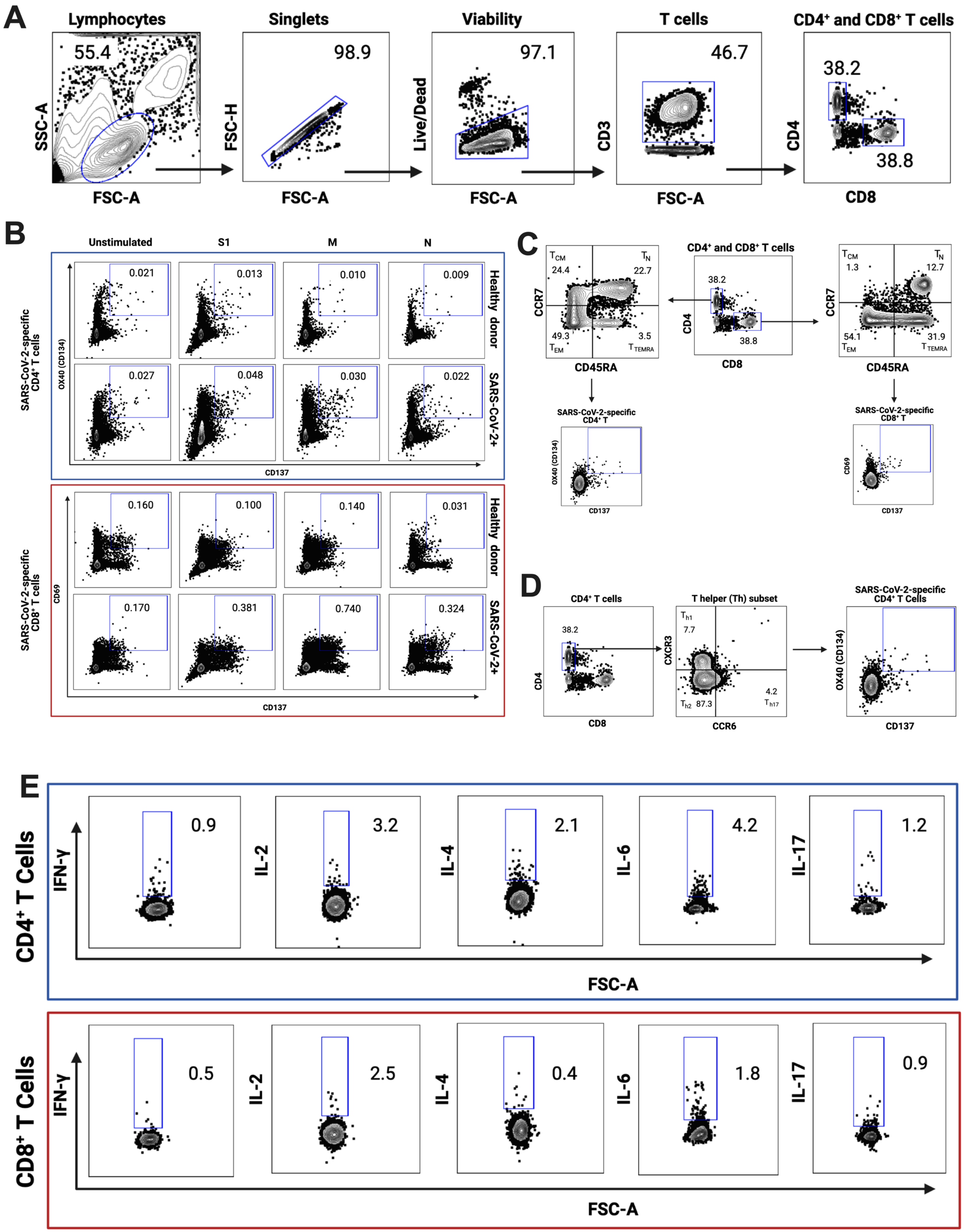

A
Lymphocytes
55.4
SSC-A
FSC-A
Singlets
98.9
FSC-H
FSC-A
Viability
97.1
Live/Dead
FSC-A
T cells
46.7
CD3
FSC-A
CD4⁺ and CD8⁺ T cells
38.2
38.8
CD4
CD8
B
Unstimulated
S1
M
N
SARS-CoV-2-specific CD4⁺ T cells
OX40 (CD134)
0.021
0.013
0.010
0.009
Healthy donor
0.027
0.048
0.030
0.022
SARS-CoV-2+
CD137
SARS-CoV-2-specific CD8⁺ T cells
CD69
0.160
0.100
0.140
0.031
Healthy donor
0.170
0.381
0.740
0.324
SARS-CoV-2+
CD137
C
CD4⁺ and CD8⁺ T cells
38.2
38.8
CD4
CD8
TCM 24.4
TN 22.7
49.3 TEM
3.5 TTEMRA
CCR7
CD45RA
TCM 1.3
TN 12.7
54.1 TEM
31.9 TTEMRA
CCR7
CD45RA
SARS-CoV-2-specific CD4⁺ T
OX40 (CD134)
CD137
SARS-CoV-2-specific CD8⁺ T
CD69
CD137
D
CD4⁺ T cells
38.2
CD4
CD8
T helper (Th) subset
Th1 7.7
Th2 87.3
4.2 Th17
CXCR3
CCR6
SARS-CoV-2-specific CD4⁺ T Cells
OX40 (CD134)
CD137
E
CD4⁺ T Cells
IFN-γ
0.9
IL-2
3.2
IL-4
2.1
IL-6
4.2
IL-17
1.2
FSC-A
CD8⁺ T Cells
IFN-γ
0.5
IL-2
2.5
IL-4
0.4
IL-6
1.8
IL-17
0.9
FSC-A

S8 Figure. Representative gating strategy used for the T cells assays

(A) T cells were defined from the gates of morphology of lymphocytes, singlets, viability and $CD3^+$. **(B)** Gating strategy used in the $CD4^+$ and $CD8^+$ T cell activation induced marker (AIM) assay to assess the SARS-CoV-2-specific $CD4^+$ and $CD8^+$ T cells after overnight stimulation with S, M and N peptide pools. Representative image comparing S-, M- and N-specific $CD4^+$ and $CD8^+$ T cells in a healthy control and a SARS-CoV-2-infected patient 6-9 months post infection. **(C)** Gating strategy used in the $CD4^+$ and $CD8^+$ T cell activation induced marker (AIM) assay to assess the SARS-CoV-2-specific memory $CD4^+$ and $CD8^+$ T cell subsets. Distribution of central memory (TCM), effector memory (TEM), and terminally differentiated effector memory cells (TEMRA) among total SARS-CoV-2–specific T cells. **(D)** Gating strategy used in the $CD4^+$ T cell activation induced marker (AIM) assay to assess SARS-CoV-2–specific T helper (Th) subsets. **(E)** Gating strategy used in the $CD4^+$ and $CD8^+$ T cell intracellular staining assay to assess the cellular cytokine profile after 6 hours stimulation with S, M and N peptide pools.

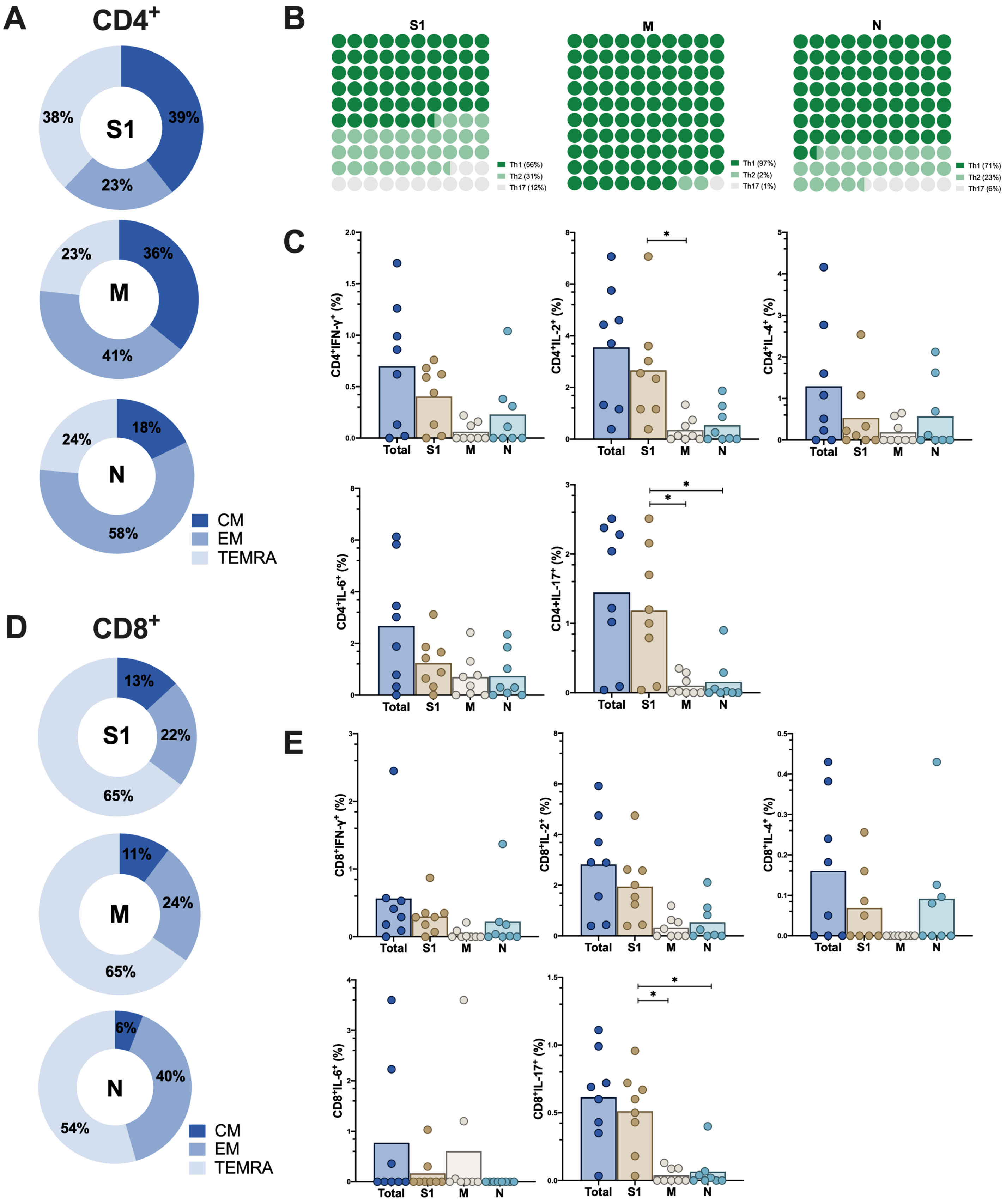
A
CD4+
S1
M
N
39%
23%
38%
36%
41%
23%
18%
58%
24%
CM
EM
TEMRA
B
S1
M
N
Th1 (56%)
Th2 (31%)
Th17 (12%)
Th1 (97%)
Th2 (2%)
Th17 (1%)
Th1 (71%)
Th2 (23%)
Th17 (6%)
C
CD4+IFN-γ+ (%)
CD4+IL-2+ (%)
CD4+IL-4+ (%)
CD4+IL-6+ (%)
CD4+IL-17+ (%)
Total
S1
M
N
*
D
CD8+
13%
22%
65%
11%
24%
65%
6%
40%
54%
E
CD8+IFN-γ+ (%)
CD8+IL-2+ (%)
CD8+IL-4+ (%)
CD8+IL-6+ (%)
CD8+IL-17+ (%)

S9 Figure. Characterization of SARS-CoV-2-specific T Cells

(A) Distribution of central memory (TCM), effector memory (TEM), and terminally differentiated effector memory cells (TEMRA) $CD4^+$ T cells targeting different proteins of SARS-CoV-2 after overnight stimulation with different peptide pools. **(B)** The $CD4^+$ Th differentiation, targeting different proteins of SARS-CoV-2, after overnight stimulation with different peptide pools. **(C)** Cytokine profile of $CD4^+$ T cells after 6 hours stimulation with S, M and N peptide pools. **(D)** Distribution of central memory (TCM), effector memory (TEM), and terminally differentiated effector memory cells (TEMRA) $CD8^+$ T cells targeting different proteins of SARS-CoV-2, after overnight stimulation with different peptide pools. **(E)** Cytokine profile of $CD8^+$ T cells after 6 hours stimulation with S, M and N peptide pools.

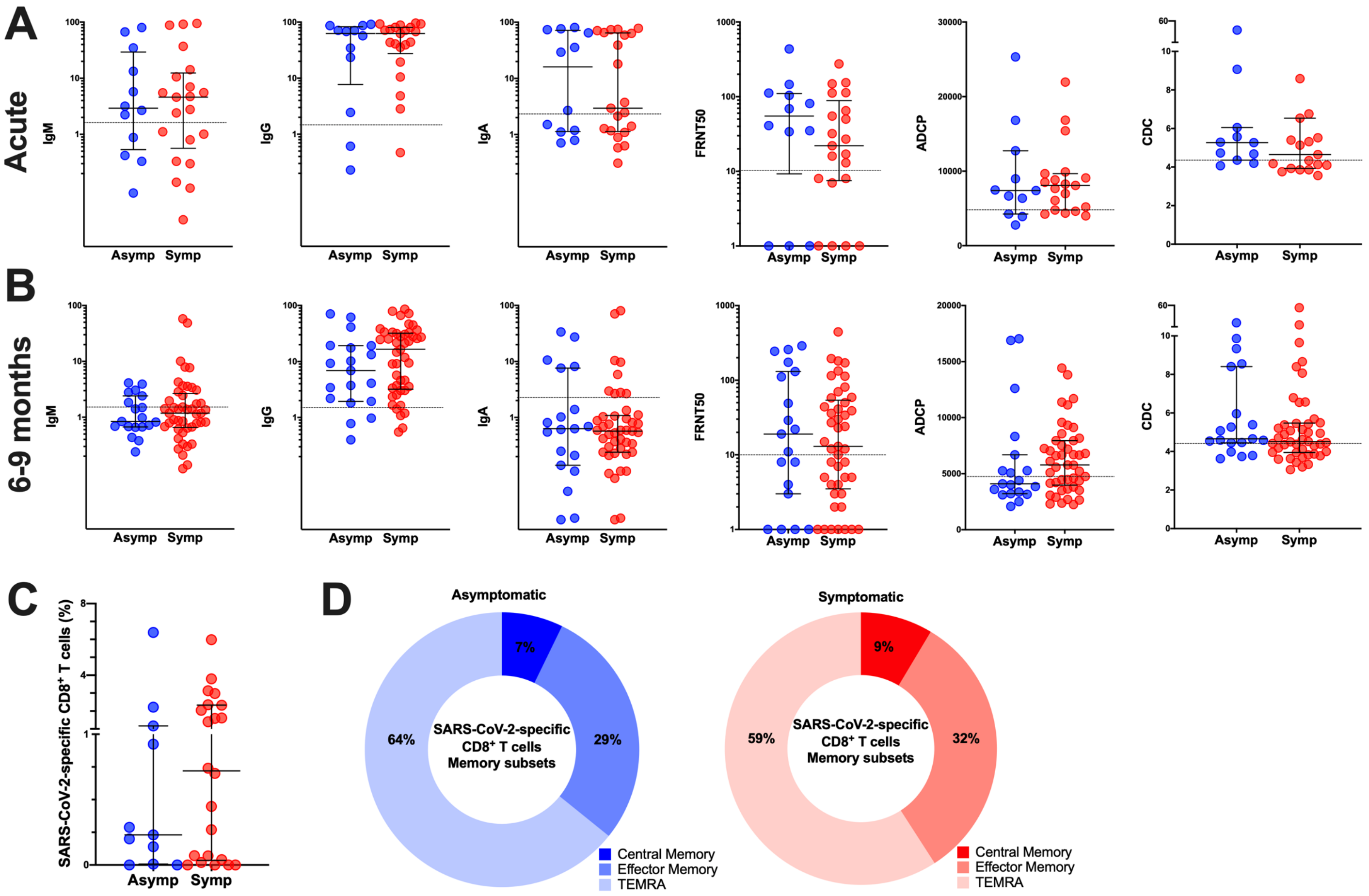

S10 Figure. Comparison of immune parameters in asymptomatic and symptomatic individuals

Comparison of anti-S antibody titers, FRNT50 titers and anti-S mediated effector functions in the **(A)** acute phase and **(B)** late convalescent phase after infection. **(C)** Comparison of the frequency of total SARS-CoV-2-specific $CD8^+$ T cells after overnight stimulation with peptide pools in asymptomatic individuals (asymp; n=11) and symptomatic patients (symp; n=22) at late convalescence. **(D)** Comparison of $CD8^+$ T cell memory phenotype between asymptomatic individuals (asymp; n=11) and symptomatic patients (symp; n=22).

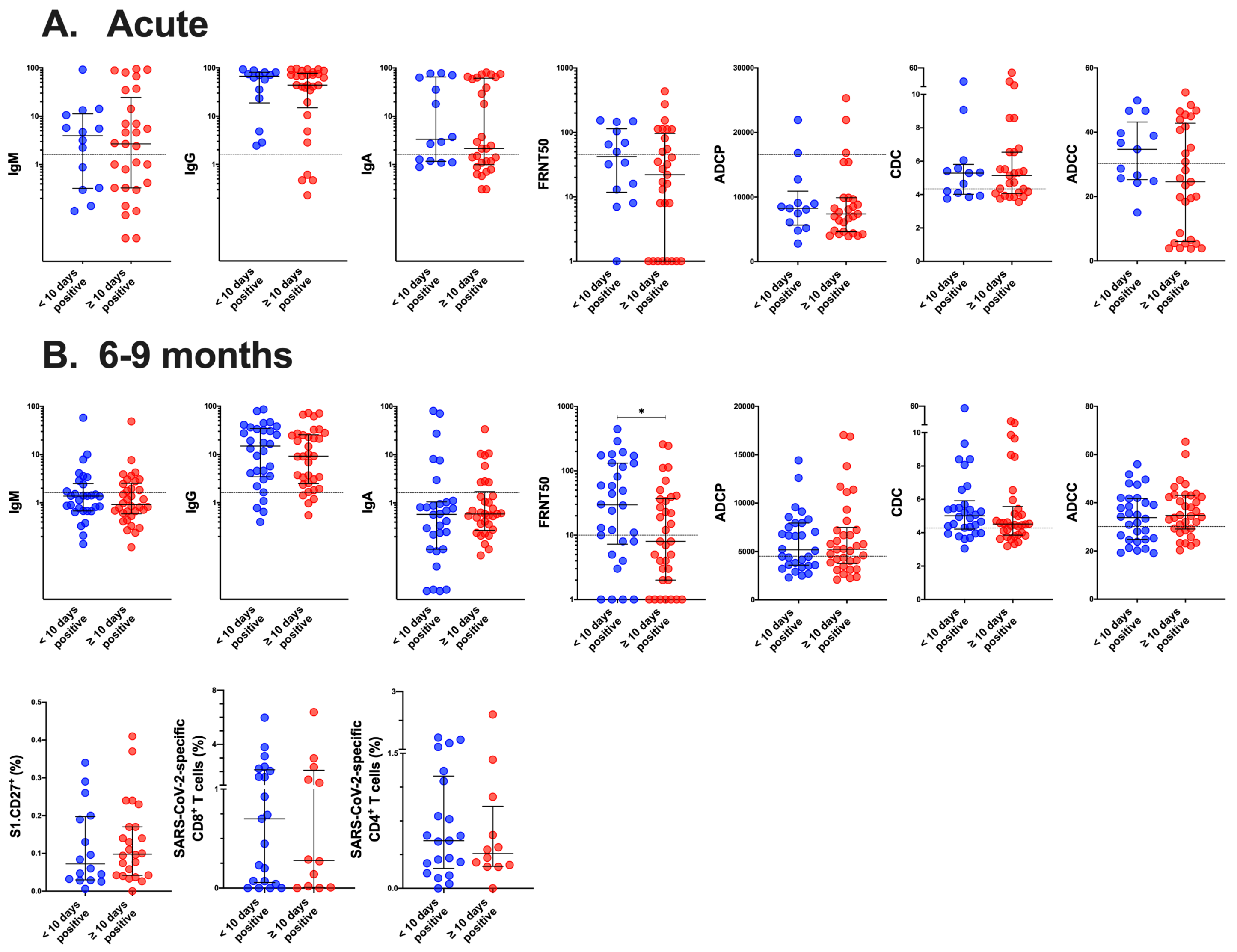

S11 Figure. Comparison of immune parameters according to time of SARS-CoV-2 shedding: < 10 days or ≥ 10 days. **(A)** Comparison of anti-S IgM, IgG and IgA antibody titers (n=33), FRNT50 titers (n=33) and anti-S mediated effector functions (ADCP and CDC n=30; ADCC n=32) in the acute phase and **(B)** comparison of anti-S IgM, IgG and IgA antibody titers (n=64), FRNT50 titers (n=64), anti-S mediated effector functions (n=64) and frequency of S1-specific CD19$^+$CD27$^+$ B cells (n=40), and total SARS-CoV-2-specific CD4$^+$ and CD8$^+$ T cells (n=33) in the late convalescent phase after infection.
